# Physicochemical Characterization and Antioxidant Activity of Humic Acids Isolated from Peat of Various Origins

**DOI:** 10.3390/molecules23040753

**Published:** 2018-03-24

**Authors:** Maria V. Zykova, Igor A. Schepetkin, Mikhail V. Belousov, Sergey V. Krivoshchekov, Lyudmila A. Logvinova, Kristina A. Bratishko, Mekhman S. Yusubov, Sergey V. Romanenko, Mark T. Quinn

**Affiliations:** 1 Department of Chemistry, Siberian State Medical University, Tomsk 634050, Russia; gmv2@rambler.ru (M.V.Z.); ludmila_logvinova@mail.ru (L.A.L.); kr-1295@mail.ru (K.A.B.); yusubov@tpu.ru (M.S.Y.); svr@tpu.ru (S.V.R.); 2 Department of Microbiology and Immunology, Montana State University, Bozeman, MT 59717, USA; schepetkin@yahoo.com; 3 Department of Pharmaceutical Analysis, Siberian State Medical University, Tomsk 634050, Russia; mvb63@mail.ru (M.V.B.); ksv_tsu@mail.ru (S.V.K.); 4 Research School of Chemical and Biomedical Technologies, Tomsk Polytechnic University, Tomsk 634050, Russia

**Keywords:** peat, humic acid, antioxidant, ethnopharmacology, semiquinone, natural products

## Abstract

Although humic acids (HAs) from peat exhibit various therapeutic properties, there is little information available concerning their physicochemical and antioxidant properties. To address this issue, nine different types of peat, including oligotrophic, mesotrophic, and minerotrophic peat samples, were used for isolation of HA fractions by basic (HAb) and pyrophosphate (HAp) extractions. Physical parameters of the HAs were analyzed by UV-Vis, fluorescent, infrared (IR), and electron paramagnetic resonance (EPR) spectroscopy. Average M_r_ of the fractions ranged from 17.2 to 39.7 kDa, while their humification index (HIX) varied from 0.49 to 1.21. HAp fractions had a higher content of aromatic structures compared to HAb fractions. Moreover, HAp fractions had a significantly higher content of phenolic OH groups (3.6 ± 0.5 mmol/g) versus HAb (3.1 ± 0.5 mmol/g). All HA fractions exhibited antioxidant activity in radical scavenging and electrochemical assays, and their EPR signal had a single line with g = 2.0035, which is consistent with semiquinone type radicals. Furthermore, the HIX was found to be important in determining the number of semiquinone-type free radicals in the HA structures. Overall, these data provide a molecular basis to explain at least part of the beneficial therapeutic properties of peat-derived HAs.

## 1. Introduction

Peat is organic soil formed as a result of incomplete disintegration and humification of marsh plants under high humidity conditions. The organic matter of peat consists of humic substances (up to 40%), lignin, polysaccharides, lipids, pectins, hemicellulose, and cellulose [1]. Humic substances are heterogeneous natural biopolymers that are contained in plant-based substances (peat, brown coal, and benthic sediment) and are represented mostly by humic acids (HAs) [1].

HAs are employed in oriental medicine, where they continue to be used extensively for treatment of skin diseases, cold stress, rheumatic pain, diabetes, kidney stones, heart ailments, leprosy, and immune system diseases [2,3]. Peat preparations are mainly used as external remedies but can also be used as internal drugs [2,4,5]. For example, HAs from various sources have been used externally for the treatment of hematoma, phlebitis, desmorrhexis, myogelosis, arthrosis, polyarthritis, osteoarthritis, and osteochondrosis [2,3]. Regarding internal use, HAs have been shown to be particular useful in therapy for diarrhea, gastritis, stomach ulcers, dysentery, colitis, and diabetes mellitus [2,3,6]. HAs have been reported in a number of studies to exhibit anti-inflammatory and immunomodulatory properties [2,3,7,8,9,10,11]. Recently, we demonstrated that peat-derived HAs exhibited antihypoxic, hepatoprotective, cardiovascular, and vasodilating activities [12,13,14,15]. Sodium humate has also been reported to be radioprotective in rats [16].

The various anti-inflammatory, immunomodulatory, and radioprotective effects of natural substances seem to be associated, at least in part, with their antioxidant and antiradical effects [17,18,19,20,21]. For example, the neuroprotective effect of HAs in a focal cerebral ischemia rat model is likely due to the antioxidant properties of HAs [22]. In renal ischemia reperfusion injury in rats, the therapeutic effects of HAs were associated with the prevention of oxidative stress [23]. HAs are protective against iron-induced hepatotoxicity and cardiotoxicity via their antioxidant and free radical scavenging effects [24]. Indeed, various humic substances are powerful antioxidants and possess reactive oxygen species (ROS) [superoxide anion (O_2_^·−^) and hydroxyl radical (OH•)] scavenging properties [25,26]. The reactivity of HAs has been attributed to the presence of phenolic and quinoid moieties [27], and HAs can behave as electron donors or acceptors, depending on the redox state of the system [27]. Thus, each HA macromolecule contains multiple antioxidant sites, a property that makes them very attractive from the environmental and biomedical point of view [28]. Considering that HAs have significant antioxidant capacity and do not have toxicity for living organisms, they have significant potential for use in the pharmaceutical or food industries [29,30,31,32].

Although antiradical and antioxidant activities of some native humic substances have been reported previously [28,32,33,34], there is no information regarding specific HAs isolated from peat. Because peat could represent an important source of HAs for the pharmaceutical industry, characterization and standardization of their physical and chemical parameters is necessary [35], especially since no clear relationships have been reported between the pharmacological activities of peat-derived HAs and their humification degree, molecular size distribution, or stable-free radical content [36]. Peat is exposed to a relatively high O_2_ levels, which leads to intensification of oxidation processes and to changes in chemical and biological characteristics of humic substances [37]. This, humification process can in turn lead to changes in pharmacological properties, such as enhancement of antiulcerogenic and antiradical activities [38]. 

The goal this work was to characterize the antiradical/antioxidant properties of HAs as a function their origin, method of isolation, and physicochemical properties. Thus, we quantified the antioxidant activity of a representative range of peat-derived HA fractions with differing physical and chemical properties.

## 2. Results and Discussion

### 2.1. Isolation of the HA Fractions

The nine peat samples analyzed varied in the degree of decomposition from 5–10% (low-mire minerotrophic samples Peat 1 and Peat 4) to 40–45% (samples of raised bog oligotrophic Peat 7 and mesotrophic Peat 9), with the ash content ranging from 2.6% (Peat 4) to 16.3% (Peat 7). The HA fractions, isolated using basic (NaOH) and pyrophosphate (Na_4_P_2_O_7_) extractions were designated as HAb1-9 and HAp1-9, respectively. The peat-derived HA fractions represent amorphous dark brown odorless powder, which is highly soluble in water. Basic extraction resulted in isolation of 1.5–3 times more HA than extraction by Na_4_P_2_O_7_ (Table 1).

### 2.2. Partial Structural Characterization of the HA Fractions

The main physicochemical parameters of the HA fractions resemble criteria developed previously for standardization of peat-derived HAs isolated for medical or biological purposes [14,35,39]. The average molecular weight (M_r_) of the HA fractions ranged from 17.2 to 39.7 kDa (Table 1), which is close to the range observed previously for soil and peat-derived HAs [40]. No difference in M_r_ was found between HAb and HAp fractions. However, HAb-1 and HAp-1 isolated from raised bog sphagnum (Peat 1) had the highest M_r_ (34.6 and 39.7 kDa), respectively, while the average M_r_ for the other 16 fractions was 21.5 ± 4.0 kDa.

The E4:E6 ratio (ratio of optical densities at 465 nm (E4) and 665 nm (E6)) in HA fractions isolated by basic extraction was significantly (*p* < 0.0001) higher than that of HA fractions obtained by pyrophosphate extraction (3.1 ± 0.19 versus 1.6 ± 0.28, respectively) (Table 1). A lower E4:E6 ratio value is associated with more abundant aromatic components and a higher degree of condensation in the structure of humic substances [41].

Infrared (IR) spectra of the HA fractions reflect the presence of typical absorption bands for peat-derived HAs [10,39] (e.g., see Supplementary Figure S1). In particular, the spectrum contained characteristic absorption bands at 3500–3300 cm^−1^ (OH stretching vibrations); 3250–3200 cm^−1^ (amide and amine N-H stretching vibrations); 2920, 2860, 1460–1440, and 700–900 cm^−1^ (stretching vibrations of –CH_3_ and –CH_2_ in side chains); 2600–2500 cm^−1^ (vibrations of carboxylic acids); 1725–1700 cm^−1^ (stretching vibrations of C=O groups that could represent ketones, aldehydes, carboxylic acids, and their functional derivatives); 1625–1610 cm^−1^ [in-plane vibrations of conjugated C-C bonds (aromatic, C=C) and C-O bonds (carbonyls bound by H-bonds, carboxylate ions, C=O) in an aromatic skeleton and quinones]; 1510–1500 cm^−1^ (uncondensed aromatic compounds bound to N and O atoms); 1250–1225 cm^−1^ [stretching (C-O) and bending (O-H) vibrations]; and 1050–1150 cm^−1^ (alcohol and carbohydrate OH stretching vibrations).

**Figure 1:**
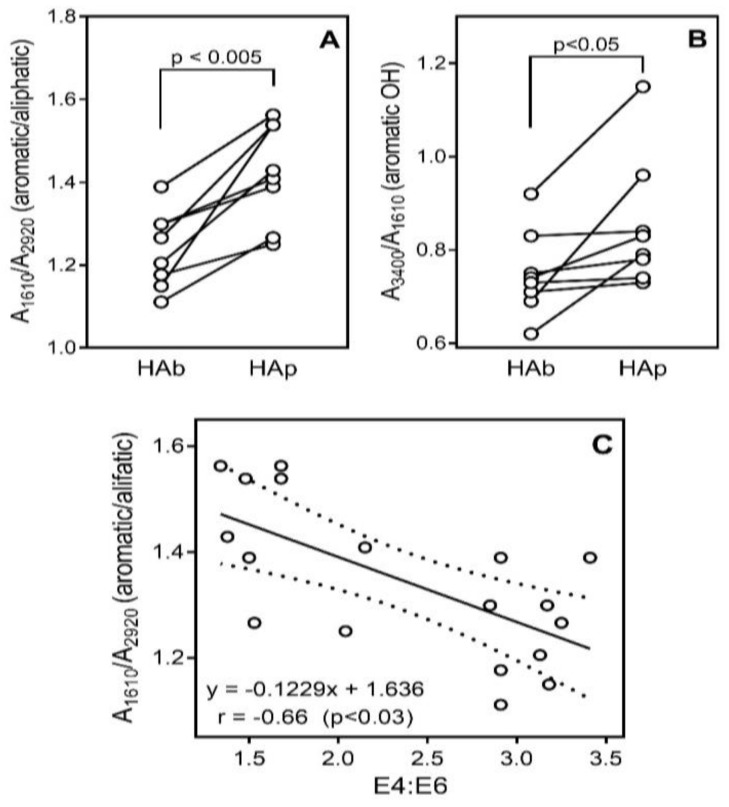
Relative concentrations of functional groups in HA fractions estimated as a ratio of intensity of the absorption bands in the IR region. A_1610_, A_2920_, and A_3400_ are optical densities for aromatic (C=C), aliphatic, and OH groups, respectively. (**Panels A**,**B**) show differences in the ratios of the absorption bands between HAb and HAp fractions. (**Panel C**) shows a plot of A_1610_/A_2920_ versus E4:E6 for all 18 HA fractions. Dashed lines indicate area of the 95% confidence band.

The method of baseline and relative densities was applied to obtain a quantitative estimate of the intensity of the absorption bands in the IR region and the relative concentrations of functional groups [39]. This analysis indicated that HAp fractions differ from HAb fractions by having a higher content of aromatic structures and phenolic (aromatic OH) groups in the HA molecules, as estimated by the A_1610_/A_2920_ and A_3400_/A_1610_ ratios, respectively (Figure 1A,B). A_1610_, A_2920_, and A_3400_ are optical densities for aromatic (C=C), aliphatic, and OH groups, respectively [42,43]. A plot of A_1610_/A_2920_ versus E4:E6 demonstrated a negative linear correlation (r = −0.66; *p* < 0.03; n = 18), supporting the conclusion that the low E4:E6 ratio is indeed associated with aromatic components [41] (Figure 1C). On the other hand, there was no definite relationship between HA extraction method and specific IR band for carboxylic groups (C=O groups, A_1720_) associated with aliphatic structures (A_2920_) in the HA molecules (data not shown).

Elemental analysis indicated that the substances isolated from peat should be classified as HAs [35]. The results showed that the HA fractions had a C content of 42.5 ± 0.6% (HAp-1) to 54.2 ± 0.68% (HAb-3), N content of 2.2 ± 0.03% (HAp-2) to 3.9 ± 0.04% (HAb-1, HAp-1, and HAb-3), H content of 4.1 ± 0.04% (HAp-7) to 6.0 ± 0.06%, and O content of 27.8 ± 0.36% to 32.8 ± 0.38% (Table 2). H/C and O/C ratios are commonly used as indicators of structural characteristics of humic substances. The generally low values for H/C in all samples indicated the presence of condensed aromatic ring structures, while the high O/C ratios may be indicative of the degree of oxygen substitution in the HA structures [44]. Indeed, a plot of H/C in all HA fractions versus E4:E6 demonstrated some linear correlation (r = 0.66; *p* < 0.005; n = 18) between these values. 

**Figure 2:**
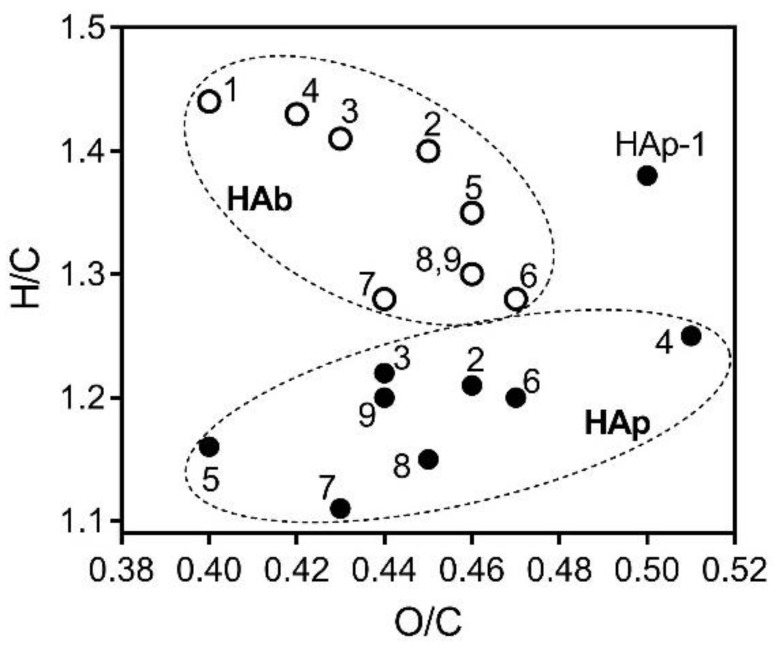
The van Krevelen diagram of H/C versus O/C for the HA fractions. HAs isolated by basic extraction are shown as open circles; HAs isolated by pyrophosphate extraction are shown as solid circles.

The van Krevelen diagram, created by plotting H/C versus O/C (Figure 2), showed that the cluster of HAp fractions was located in a region with lower H/C values compared to the cluster of HAb fractions, indicating a higher content of aromatic and/or condensed aromatic molecules [45,46], which is supported by the UV-Vis (E4:E6) and IR (A_1610_/A_2920_) spectroscopy analyses of these fractions. Notably, HAb and HAp isolated from low-mire peat samples with a high degree of decomposition (Peat 5–9) were characterized by more aromatic structures compared to HA fractions obtained from raised bog peat samples (Peat 1–4), which is consistent with our previous publications [11,39]. The HAb fractions were mainly aliphatic in nature and were comprised mainly of molecules having H/C values centered at 1.3–1.4 [47]. There was no difference in the average O/C values of the HAb and HAp fractions, which is indicative of their similar carbohydrate content, carboxylic groups, and degree of oxidation.

**Figure 3:**
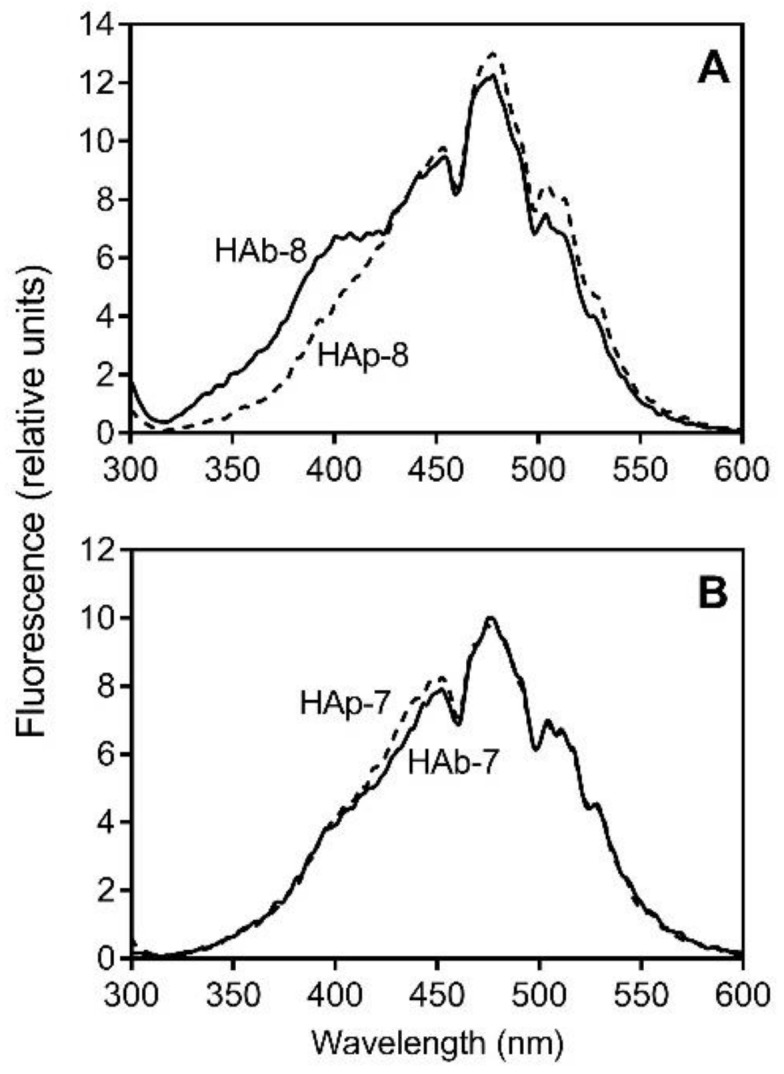
Synchronous fluorescence spectra of selected HA fractions. Solutions of HAb-8 and HAp-8 (**Panel A**) and solutions of HAb-7 and HAp-7 (**Panel B**) (10 μg/mL in 25 mM NaHCO_3_) were analyzed with a scanning fluorometer, and the synchronous spectra (Δλ = 20 nm) are shown.

To improve peak resolution compared with the conventional emission fluorescence technique, we used synchronous-scan fluorescence analysis [48]. In general, synchronous-scan fluorescence spectra of the 18 HA fractions reflected features of soil HAs [49]. However, we found that the spectra of HAb fractions had an additional fluorescence peak at 390–410 nm, except for HA fractions isolated from low-mire Peat 6 and 7. For example, the fluorescence spectra of the HAb-7–HAp-7 and HAb-8–HAp-8 pairs are shown in Figure 3. 

Fluorescence spectrometry was also used to determine the extent of humification in the peat HAs [50,51]. The humification index (HIX) ranged from 0.49 to 1.21 in the HA samples (Table 1) and indicates the degree of complexity and condensed (aromatic) nature of the fractions [50,51]. The linear correlation coefficients between E4:E6 and HIX were not statistically significant (*p* > 0.05).

### 2.3. Content of Carboxylic and Phenolic Groups in the HA Fractions

Carboxylic and phenolic groups are the most important ionizable sites present in HAs and are likely to determine many of the proton binding properties of these substances [52]. We analyzed the content of these groups by chemical titration. According to the values obtained for total acidity of the peat HAs (Table 3), 50% to 64% of the HA acidic functional groups can be attributed to phenolic OH groups. 

**Figure 4:**
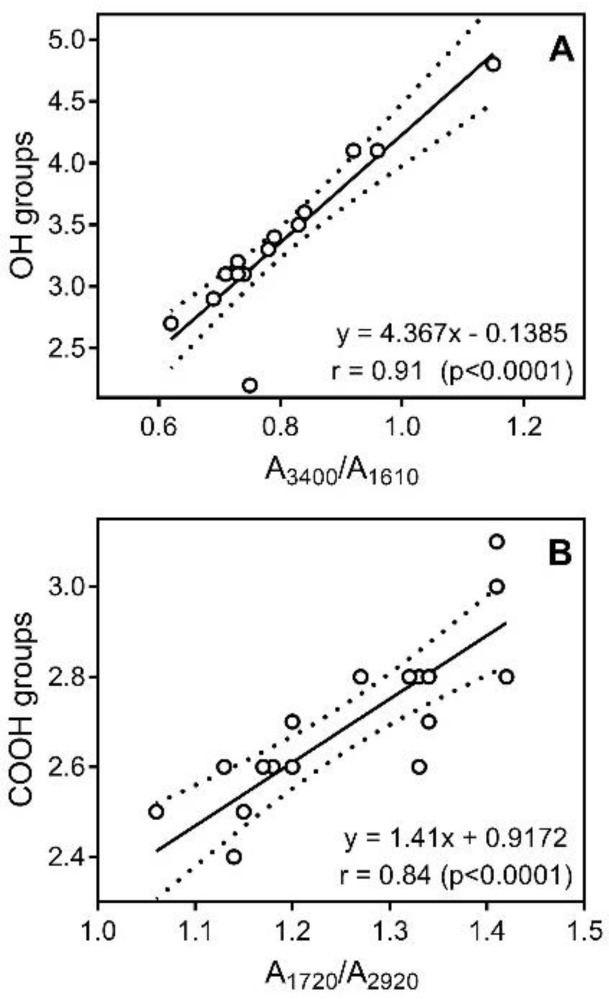
A plot of content of aromatic OH groups (**Panel A**) and COOH groups (**Panel B**) in the HA fractions versus ratio of intensity of the absorption bands in the IR region specific for OH groups (A_3400_/A_1610_) (**Panel A**) and COOH groups (A_1720_/A_2920_) (**Panel B**). Dashed lines indicate area of the 95% confidence band. Note that the symbols for HAb-4, HAb-6, HAp-6, HAb-8, and HAp-8 overlap in (**Panel A**), and the symbols for HAb-1 and HAb-3 overlap in (**Panel B**).

These data correlate well with our IR analysis, specifically with the A_3400_/A_1610_ and A_1720_/A_2920_ ratios for OH and COOH groups, respectively (Figure 4). We found that HAp fractions have significantly (*p* < 0.02) higher content of phenolic OH groups (3.6 ± 0.5 mmol/g) compared to HAb (3.1 ± 0.5 mmol/g). However, HAb and HAp showed no significant differences in their average content of COOH groups (2.6 ± 0.1 mmol/g versus 2.7 ± 0.2 mmol/g, respectively).

### 2.4. Antioxidant Activity of the HA Fractions

Antioxidant activity of the HA samples was evaluated in electrochemical reduction and 1,1-diphenyl-2-picrylhydrazyl (DPPH) radical-scavenging assays. Electrochemical O_2_ reduction at the mercury film electrode proceeds at the cathode in several stages, with formation of O_2_^·−^ and H_2_O_2_. The voltammetric methodology allows evaluation of the antioxidant activity of natural compounds and, moreover, consideration of their influence on the kinetics of the electrochemical O_2_ reduction can suggest mechanisms of interaction of the compounds with ROS in model systems [53]. We found that the antioxidant activity of HAp fractions was higher than that of the HAb fractions, with two exceptions being the HA fractions isolated from raised bog peat (Peat 1 and 3) (Table 4).

**Figure 5:**
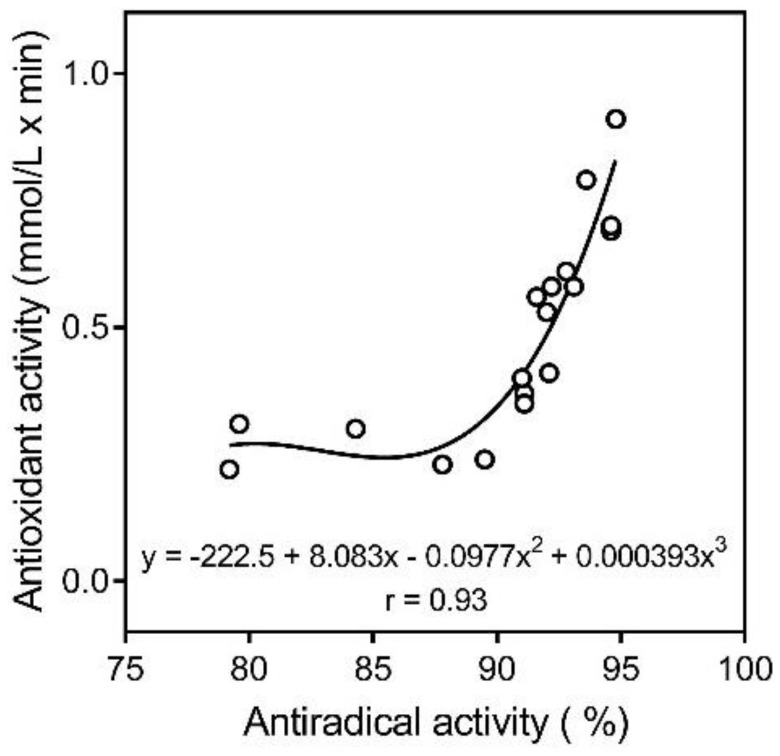
Polynomial extrapolation of antiradical activity in the DPPH assay versus antioxidant activity in the electrochemical assay for all 18 HA fractions.

To determine free radical scavenging activity of the HA fractions, we evaluated reaction with DPPH (Table 4). Additionally, nonlinear (polynomial) extrapolation performed on plots of antiradical activity in the DPPH assay versus antioxidant activity in electrochemical assay demonstrated a good correlation coefficient (r = 0.93) (Figure 5).

Interaction of the proton of these oxygen-containing functional groups in the structure of HAs (HA–H) with the active free radical DPPH• is responsible for the ability of humic substances to bind free radicals in the system and to remove active radical species from the reaction sphere, according to the following reaction [34]:

HA–H + DPPH•→HA• + DPPH–H(1)

The antioxidant/antiradical activity of HAs is attributed to the presence of semiquinone-type radicals in their structure [54], and the stability of these radicals is sustained by the condensed aromatic structures in HA [55]. We found that the electron paramagnetic resonance (EPR) signal of all HA fractions exhibited a single line with g = 2.0035 ± 0.0002, which is consistent with the presence of semiquinone-type radicals [56] (e.g., see Supplementary Figure S2). The total amount of the semiquinone-type free radicals in each HA fraction was evaluated by EPR as the number of paramagnetic centers (PMC) (Table 4). Note, however, that we did not find a significant difference in the average PMC content in HAb versus HAp fractions (7.8 ± 1.6 and 8.5 ± 4.1 spin/g × 10^−16^, respectively).

**Figure 6:**
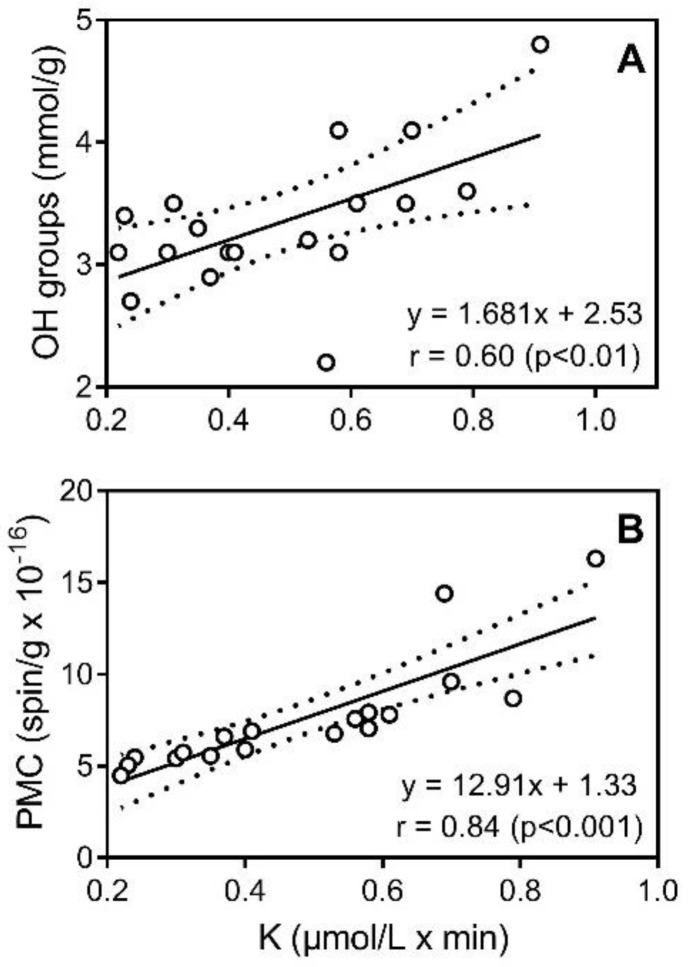
Plot of aromatic OH group content (**Panel A**) and number of paramagnetic centers (PMC) (**Panel B**) in the HA fractions versus antioxidant activity of the fractions in the electrochemical assay for all 18 HA fractions. Dashed lines indicate area of the 95% confidence band.

**Figure 7:**
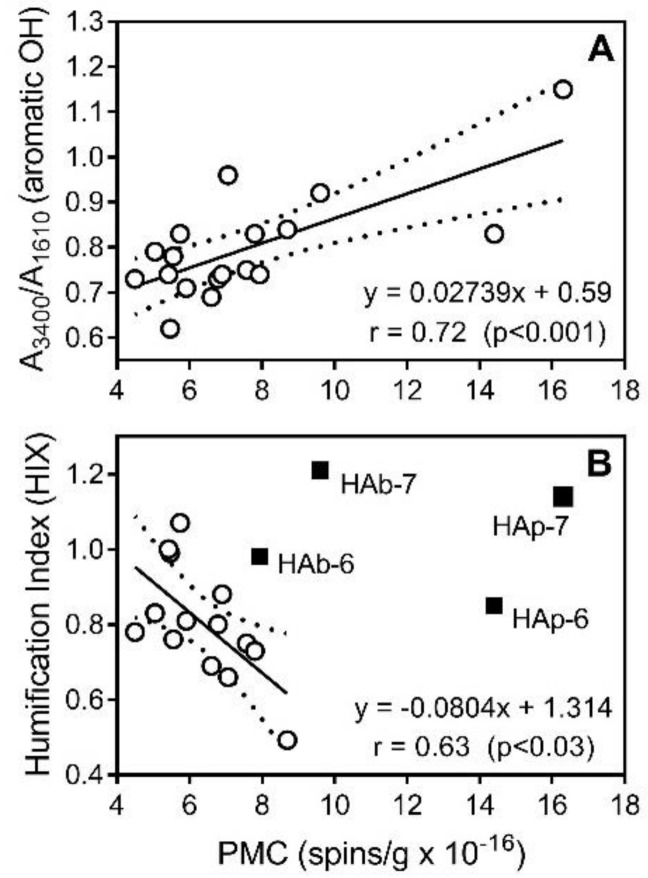
Plot of the number of paramagnetic centers (PMC) versus relative content of aromatic OH groups (by IR spectra) (**Panel A**) and HIX values (**Panel B**) for all 18 HA fractions. Dashed lines indicate area of the 95% confidence band.

HAs contain a wide variety of moieties that are oxidized at different potentials. When they are oxidized, they release protons and electrons and undergo irreversible follow-up reactions [27]. For example, electron and hydrogen-donating phenolic antioxidants can react with ROS in a termination reaction [27,57]. The activity of many natural compounds toward DPPH is due to the presence of a variety of phenol fragments and correlates with the total concentration of OH groups [58]. Indeed, Aeschbacher et al. [27] reported that phenolic moieties are the major electron donating groups in a humic substances. On the other hand, the number of quinoid moieties account for only part of the HA structural fragments involved in electron transfer. Apart from quinoid/semiquinoid moieties, phenolic groups oxidized to phenoxyl radicals can also contribute to antioxidant activity of HAs [33,59]. The EPR and chemical titration analyses show that higher amounts of PMC and phenolic OH groups in the HA structure improves antioxidant activity, with significant coefficients of linear correlation r = 0.60 (*p* < 0.01) and r = 0.84 (*p* < 0.001), respectively (Figure 6). It should be noted that HAp fractions from low-mire grass peat samples (Peat 6 and 7) have the highest number of PMC and antioxidant activities (Table 4). Moreover, in accordance with Schnitzer and Levesque [60], we found that the number of PMC correlated significantly (r = 0.72, *p* < 0.001) with the concentration of phenolic OH groups (Figure 7A). No correlation was found between the content of carboxylic groups and antiradical activity (*p* < 0.05; r = 0.09) or antioxidant activity (*p* < 0.05; r = 0.11). Thus, although a strong correlation was previously reported between complement-fixing activity and carboxylic group content in some fractions of humic substances [8], the carboxylic groups probably do not play an important role in antiradical/antioxidant activity of peat-derived HAs.

In the present study, the negative correlation between HIX and number of PMC was found for most of HAs, with the exception of fractions derived from low-mire samples (Peat 6 and 7; Figure 7B). According to Rosa et al. [61], phenolic groups formed during organic matter decomposition undergo oxidation, producing quinone- and hydroquinone-type structures. These structures, which are predecessors of semiquinone-type free radicals, can presumably increase as the state of humification advances and should be associated with increasing HIX. Indeed, a positive relationship was reported between HIX obtained using laser-induced fluorescence spectroscopy (LIFS) and the number of semiquinone radicals [62]. Conversely, quinone- and hydroquinone-type structures have their own fluorescence and could affect the optic parameters measured for HAs. Moreover, reduction of HAs could be accompanied by a blue-shift in their fluorescence spectrum [63], which may be due to the increase of hydroquinone/quinone-groups and decreasing HIX calculated as Σ*I*_435__→480_)/Σ*I*_300__→345_ (see *Methods*).

In conclusion, peat-derived HAp fractions differ from HAb mainly in the higher content of condensed and/or aromatic structures and phenolic groups. A high number of semiquinone-type radicals and antioxidant/antiradical activity were found in HA fractions isolated from low-mire peat samples (sampling at > 2 m depth of a peat column). Thus, the high level of antioxidant activity of low-mire peat HAp samples may contribute to their beneficial therapeutic properties, and future research on this issue is warranted.

## 3. Materials and Methods

### 3.1. Isolation of HAs from Peat

For HA isolation, nine representative types of peat were taken from major peat bogs in the Tomsk region of Russia, including the oligotrophic bog located in southern taiga zone between the Iksa and Bakchar rivers and representing the northeastern spurs of the Great Vasyugan Mire [64] (56°58′ N latitude and 82°36′ W longitude; Peat 1–4, 6, 7, and 9) and the eutrophic bogs Klyukvennoe (56°23′ N latitude and 84°42′ W longitude; Peat 5) and Tagan (56°21′ N latitude and 84°48′ W longitude; Peat 8). Different peat profiles in the bogs were chosen, and samples were taking from different depths of the peat sections. The degree of decomposition of the peat samples was analyzed microscopically (at 56–140× magnification) as a percentage of groundmass to volumetric amount of total tissues in the peat samples. For isolation of HAs, the peat was desiccated at room temperature, milled, and treated with 0.1 M Na_4_P_2_O_7_ (pyrophosphate extraction) or 0.1 M NaOH (basic extraction) [48] for 5–8 h with constant mixing at 30–50 °C. The sediment was separated from the fluid by filtration. For HA precipitation from the solution, the fluid was treated with HCl at pH 1.0–2.0, and the isolated HAs were separated by centrifugation, washed on a filter with water up to pH 7.0, and dried at room temperature. Yield of HAs from the peat samples was measured by the gravimetric method.

### 3.2. Physical Characterization of HAs

Infrared (IR) absorption spectra of the isolated HAs were recorded using an Fourier transform IR spectrometer (FSM 1201, Infraspek Ltd., St. Petersburg, Russia) in KBr tablets at a ratio of 1:100 and at 500–4000 cm^−1^. Electronic absorption spectra of the aqueous solutions of HAs (10 µg/mL) in quartz cuvettes (1 cm) were recorded on a Unico 2800 spectrophotometer (Dayton, NJ, USA) at 190–700 nm. The optical densities at 465 nm (E4) and 665 nm (E6) were determined from the spectra for calculation of the E4:E6 ratio. The elemental composition was determined by combustion on a Carlo Erba Strumentazione Model 1106 C, H, N analyzer (Milan, Italy), and the O_2_ content was determined as the difference. The concentration of semiquinone-type free radicals in solid-state samples was determined as the number of PMC by EPR spectroscopy [55] using an EMX EPR spectrometer (Bruker, Rheinstetten, Germany) at room temperature (~23 °C). While determining the absolute concentration of unpaired spins, CuCl_2_·5H_2_O served as a standard containing a known number of PMC.

Fluorescence measurements were performed using an LS50B luminescence spectrometer (Perkin Elmer, Norwalk, CT, USA). The samples were dissolved in NaHCO_3_ (25 mM, pH 8.5). The slit width for emission and excitation wavelengths was 10 nm. The HIX was determined using the formula: HIX = (Σ*I*_435__→480_)/(Σ*I*_300__→345_), where *I* is the fluorescence emission intensity with excitation at 254 nm [51]. Since fluorescence intensity can be attenuated by the solution itself (i.e., inner-filtering effect), both primary and secondary fluorescence inner-filtering effects were corrected for in order to obtain an accurate measurement of the fluorescence emission intensity. For calculation of HIX values corrected for inner-filter effects, linear extrapolation was performed on plots of HIX versus transmittance at 254 nm for 6–7 different concentrations of each fraction. The corrected HIX values correspond to infinite dilution (i.e., approximating 100% transmittance). Synchronous fluorescence spectra were recorded from 250 to 600 nm at a scan rate of 240 nm/min. The excitation–emission wavelength difference (Δλ) was 20 nm [48].

### 3.3. High Performance Size-Exclusion Chromatography (HP-SEC)

Average molecular weights of the HA fractions were determined by HP-SEC using Ultrahydrogel 250 column (300 × 7.8 mm, 6 µm, pore size 250 Å; Waters, Milford, MA, USA). The mobile phase was 0.1 M Tris-HCl, pH 8.9 (1 mL/min). We used an Ultimate 3000 chromatograph (Dionex, Sunnyvale, CA, USA) equipped with a vacuum degasser, LPG-3400SD pump, column thermostat TCC-3000SD, and a spectrophotometric detector DAD-3000 operating at 240 nm. The molecular weights of the fractions were estimated by comparison with the retention times of polystyrene sulfonate standards (PSS Polymer Standards Service GmbH, Mainz, Germany).

### 3.4. Chemical Analysis of HAs

In order to measure total acidity, the HA samples were treated with a Ba(OH)_2_ solution under N_2_ atmosphere for 24 h. The Ba(OH)_2_ remaining in the solution after the reaction was then back-titrated with a standard acid solution. For the titration of carboxylic acid groups, the HA samples were treated for 24 h with calcium acetate solution in excess, which causes the release of acetic acid. The CH_3_COOH released was then titrated with a standard base solution. Phenolic OH groups were calculated as the difference between total acidity and acidity of the carboxylic groups [65]

### 3.5. Free Radical Scavenging Activity

The antioxidant activity of the HA fractions was determined by measuring their capacity to bleach the purple-colored methanol solution of DPPH, which represents a suitable basis for comparative evaluation of the radical scavenging activity for most natural antioxidants [66,67]. In brief, a methanolic solution of DPPH (254 µmol/L) was mixed in a 1-cm cuvette with each HA sample (1 μg/mL) at room temperature. The decrease in absorbance at 520 nm was monitored for 60 min using a Unico 2800 spectrophotometer. For comparison, similar kinetic experiments were also performed for DPPH interacting with buffer alone (negative control). The radical scavenging activity is expressed as percent inhibition, and all determinations were performed in triplicate.

### 3.6. Electrochemical Experiments

Antioxidant activity of the HA samples was evaluated by measuring the electrochemical reduction current of O_2_ at a mercury film electrode after reaction of the compounds with O_2_^·−^ [53,68]. A phosphate buffer solution (pH 6.9) served as the supporting electrolyte. HA fractions were placed in an electrochemical cell at a final concentration of 1 µg/mL and stirred. Voltammograms of the cathodic reduction of O_2_ were recorded with an AOA voltammetric analyzer (Tomsk, Russia) using linear sweep voltammetry under the following conditions: potential scan rate 30 mV/s and potential range E = 0 to −0.7 V. The electrochemical cell consisted of a working mercury film electrode, a silver/silver chloride reference electrode with saturated KCl. It should be noted that the HAs were not adsorbed to the surface of the working mercury film electrode in the range of the O_2_ reduction [53]. Test compounds reacted with ROS and changed the electrochemical reduction current of O_2_ (first wave at E = −0.3 V). The voltammograms were used to plot time dependences of the function [1–*I*/*I*_0_] in the presence of a test sample. The linear part of the plot and the slope ratio of the tangent to this portion of the curve were used to calculate the kinetic criterion of antioxidant activity, K (μmol/L × min^−1^), of a sample using the formula:

(2) $$K=\frac{C_{O_{2}}}{t}\left(1-\frac{I_{i}}{I_{o}}\right)$$

where $C_{O_{2}}$ is the initial O_2_ concentration in the solution (μmol/L), *t* is the duration of the interaction between the test substance and ROS (min), *I_i_* is the change of the O_2_ reduction current density after addition of a test sample (μA), and *I_o_* is the limiting current of the O_2_ reduction in the absence of the substance being analyzed (μA). All determinations were performed in triplicate.

### 3.7. Statistical Analysis

Linear regression analysis was performed on the indicated sets of data to obtain correlation coefficients, 95% confidence intervals, and statistical significance (GraphPad Prism Software, San Diego, CA, USA). The data pertaining to physicochemical characteristics and content of carboxylic and phenol groups in different HA fractions were also analyzed using two-way analysis of variance (ANOVA). Both linear regression and ANOVA were considered statistically significant at *p* < 0.05.

## Figures and Tables

**Table 1:** Characterization of the peat samples, yields of HA fractions isolated by basic and pyrophosphate extraction from the peat samples, and selected physicochemical properties.

Peat Type, Sample Name, Sampling Depth (cm), and Degree of Decay (%)	Fraction	Yield (%)	E4:E6	HIX	M_r_ (kDa)
Raised bog sphagnum Peat 1, 20–70 cm, 5–10%	HAb-1	6.5 ± 0.1	2.85 ± 0.05	0.99	34.6
	HAp-1	3.1 ± 0.1 ^a^	1.50 ± 0.02 ^b^	0.83	39.7
Raised bog pine-cotton-grass, Peat 2, 10–50 cm, 30–35%	HAb-2	31.4 ± 0.2	2.91 ± 0.02	0.69	19.0
	HAp-2	13.2 ± 0.1 ^a^	2.04 ± 0.02 ^b^	0.66	22.8
Raised bog magellanicum, Peat 3, 20–70 cm, 10–15%	HAb-3	16.9 ± 0.1	3.17 ± 0.01	0.75	27.8
	HAp-3	4.2 ± 0.1 ^a^	2.15 ± 0.01 ^b^	0.76	18.8
Raised bog fuscum, Peat 4, 20–70 cm, 5–10%	HAb-4	13.3 ± 0.1	2.91 ± 0.02	0.73	31.4
	HAp-4	3.9 ± 0.1 ^a^	1.53 ± 0.02 ^b^	0.49	25.8
Low-mire woody, Peat 5, 10–50 cm, 25–30%	HAb-5	38.2 ± 0.4	2.91 ± 0.03	0.81	24.4
	HAp-5	26.0 ± 0.3 ^a^	1.68 ± 0.04 ^b^	0.80	21.2
Low-mire grass-moss, Peat 6, 200–250 cm, 35–50%	HAb-6	21.5 ± 0.2	3.41 ± 0.02	0.98	22.1
	HAp-6	6.8 ± 0.1 ^a^	1.34 ± 0.01 ^b^	0.85	20.9
Low-mire grass, Peat 7, 230–270 cm, 40–45%	HAb-7	37.3 ± 0.4	3.25 ± 0.06	1.21	20.8
	HAp-7	17.4 ± 0.6 ^a^	1.48 ± 0.01 ^b^	1.14	16.7
Low-mire woody peat, Peat 8, 50–100 cm, 30–35%	HAb-8	38.6 ± 0.1	3.18 ± 0.07	1.01	17.6
	HAp-8	17.9 ± 0.1 ^a^	1.68 ± 0.01 ^b^	1.07	17.2
Mesotrophic carex peat, Peat 9, 150–200 cm, 40–45%	HAb-9	27.3 ± 0.1	3.13 ± 0.05	0.78	20.0
	HAp-9	8.0 ± 0.1 ^a^	1.38 ± 0.01 ^b^	0.88	17.5

Significant differences (^a^, *p* < 0.001; ^b^, *p* < 0.05) between fractions isolated by basic (HAb) and pyrophosphate (HAp) extraction from the same peat samples are indicated.

**Table 2:** Elemental composition of the HA fractions expressed as weight percent.

Fraction	С	Н	N	О
HAb-1	53.3 ± 0.61	6.0 ± 0.06	3.9 ± 0.04	28.6 ± 0.39
HAp-1	42.5 ± 0.61 ^a^	4.6 ± 0.05 ^a^	3.9 ± 0.04	28.5 ± 0.38
HAb-2	52.2 ± 0.64	5.6 ± 0.05	2.8 ± 0.03	31.5 ± 0.36
HAp-2	52.2 ± 0.65	4.8 ± 0.05 ^a^	2.2 ± 0.03 ^a^	31.6 ± 0.39
HAb-3	54.2 ± 0.68	6.0 ± 0.06	3.9 ± 0.05	30.9 ± 0.45
HAp-3	51.5 ± 0.64 ^a^	4.8 ± 0.05 ^a^	3.4 ± 0.04 ^a^	30.1 ± 0.40
HAb-4	53.9 ± 0.69	6.0 ± 0.05	3.5 ± 0.04	30.0 ± 0.45
HAp-4	49.3 ± 0.62 ^a^	4.7 ± 0.05 ^a^	3.2 ± 0.04	33.6 ± 0.44 ^a^
HAb-5	51.8 ± 0.74	5.4 ± 0.05	3.5 ± 0.03	31.5 ± 0.38
HAp-5	52.5 ± 0.65	4.6 ± 0.05 ^a^	3.1 ± 0.04 ^a^	27.8 ± 0.36 ^a^
HAb-6	52.3 ± 0.65	5.1 ± 0.05	3.4 ± 0.05	32.8 ± 0.38
HAp-6	46.4 ± 0.61 ^a^	4.2 ± 0.04 ^a^	3.2 ± 0.03	29.3 ± 0.38 ^a^
HAb-7	49.2 ± 0.62	4.8 ± 0.05	3.6 ± 0.04	29.0 ± 0.46
HAp-7	49.5 ± 0.62	4.1 ± 0,04 ^a^	3.3 ± 0.03	28.5 ± 0.35
HAb-8	51.7 ± 0.57	5.4 ± 0.05	3.8 ± 0.03	32.1 ± 0.39
HAp-8	52.1 ± 0.65	4.5 ± 0.04 ^a^	3.1 ± 0.03 ^a^	31.0 ± 0.42
HAb-9	52.7 ± 0.56	5.3 ± 0.06	3.0 ± 0.03	32.0 ± 0.38
HAp-9	51.9 ± 0.63	4.7 ± 0,05 ^a^	3.1 ± 0,03	30.6 ± 0.43

All elemental contents are expressed on an ash-free basis. ^a^ Significant differences (*p* < 0.05) between fractions isolated by basic (HAb) and pyrophosphate (HAp) extraction from the same peat samples are indicated.

**Table 3:** Acidic functional groups of the HA fractions determined by chemical titration.

Fraction	Acid Groups (mmol/g)		
	СООН	ОН_phenolic_	Total Acidity
HAb-1	2.5 ± 0.01	2.7 ± 0.03	5.2 ± 0.06
HAp-1	2.4 ± 0.01	3.4 ± 0.02 ^a^	5.7 ± 0.03 ^a^
HAb-2	2.8 ± 0.01	2.9 ± 0.01	5.8 ± 0.02
HAp-2	2.7 ± 0.01	4.1 ± 0.02 ^a^	6.8 ± 0.04 ^a^
HAb-3	2.4 ± 0.01	2.2 ± 0.03	5.7 ± 0.06
HAp-3	2.8 ± 0.01 ^a^	3.3 ± 0.02 ^a^	6.1 ± 0.03 ^a^
HAb-4	2.6 ± 0.01	3.5 ± 0.03	6.1 ± 0.06
HAp-4	2.5 ± 0.01	3.6 ± 0.02	6.1 ± 0.03
HAb-5	2.8 ± 0.01	3.1 ± 0.02	5.9 ± 0.03
HAp-5	2.8 ± 0.01	3.2 ± 0.03	6.0 ± 0.05
HAb-6	2.8 ± 0.01	3.1 ± 0.01	5.9 ± 0.01
HAp-6	2.6 ± 0.01 ^a^	3.5 ± 0.02 ^a^	6.2 ± 0.03 ^a^
HAb-7	2.6 ± 0.01	4.1 ± 0.03	6.7 ± 0.06
HAp-7	2.7 ± 0.01	4.8 ± 0.03 ^a^	7.5 ± 0.06 ^a^
HAb-8	2.6 ± 0.01	3.1 ± 0.02	5.7 ± 0.03
HAp-8	3.0 ± 0,01 ^a^	3.5 ± 0.02 ^a^	6.5 ± 0.04 ^a^
HAb-9	2.6 ± 0,01	3.1 ± 0.01	5.7 ± 0.01
HAp-9	3.1 ± 0.01 ^a^	3.1 ± 0.01	6.2 ± 0.02 ^a^

^a^ Significant differences (*p* < 0.05) between fractions isolated by basic (HAb) and pyrophosphate (HAp) extraction from the same peat samples are indicated.

**Table 4:** Analysis of antioxidant activities of the HA fractions using voltammetry and DPPH assays and determination of the number of paramagnetic centers (PMC) in each fraction.

Fraction	Voltammetry (µmol/L × min^−1^)	DPPH (% Inhibition)	PMC (spin/g × 10^−16^)
HAb-1	0.24 ± 0.05	89.5 ± 0.02	5.47
HAp-1	0.23 ± 0.03	87.8 ± 0.14 ^a^	5.05
HAb-2	0.37 ± 0.05	91.1 ± 0.20	6.60
HAp-2	0.58 ± 0.06 ^a^	92.2 ± 0.12	7.06
HAb-3	0.56 ± 0.03	91.6 ± 0.22	7.57
HAp-3	0.35 ± 0.04 ^a^	91.1 ± 0.20	5.55
HAb-4	0.61 ± 0.05	92.8 ± 0.26	7.8
HAp-4	0.79 ± 0.06 ^a^	93.6 ± 0.15	8.69
HAb-5	0.40 ± 0.05	91.0 ± 0.20	5.91
HAp-5	0.53 ± 0.02 ^a^	92.0 ± 0.23	6.78
HAb-6	0.58 ± 0.06	93.1 ± 0.27	7.93
HAp-6	0.69 ± 0.07 ^a^	94.6 ± 0.18	14.4
HAb-7	0.70 ± 0.02	94.6 ± 0.25	9.6
HAp-7	0.91 ± 0.05 ^a^	94.8 ± 0.18	16.3
HAb-8	0.30 ± 0.02	84.3 ± 0.01	5.42
HAp-8	0.31 ± 0.04	79.6 ± 0.12 ^a^	5.74
HAb-9	0.22 ± 0.02	79.2 ± 0.11	4.49
HAp-9	0.41 ± 0.05 ^a^	92.1 ± 0.14 ^a^	6.9

^a^ Significant differences (*p* < 0.05) between fractions isolated by basic (HAb) and pyrophosphate (HAp) extractions from same peat samples are indicated.

## Supplementary Materials

Supplementary Materials are avaliable online.
