# Supplementary material for: Physicochemical Characterization and Antioxidant Activity of Humic Acids Isolated from Peat of Various Origins
Source: Molecules. 2018 Mar 24;23(4):753. doi: 10.3390/molecules23040753 (PMC6017172; doi:10.3390/molecules23040753)

Supplementary Material

Physicochemical Characterization and Antioxidant Activity of Humic Acids Isolated from Peat of Various Origins

Maria V. Zykova [1,†], Igor A. Schepetkin [2,†], Michael V. Belousov [1], Sergey V. Krivoshchekov [1,3], Lyudmila A. Logvinova [1], Kristina A. Bratishko [1], Mekhman S. Yusubov [1,3], Sergey V. Romanenko [1,3] and Mark T. Quinn [2,*]

[1] Siberian State Medical University, Tomsk, 634050, Russia
[2] Department of Microbiology and Immunology, Montana State University, Bozeman, MT 59717, USA
[3] Tomsk Polytechnic University, Tomsk, 634050, Russia
† These authors contributed equally to this work.
* Correspondence: mquinn@montana.edu; Tel.: +1-406-994-4707; Fax: 1-406-994-4303

Supplementary Figure S1. Representative IR spectra of HAp-7.

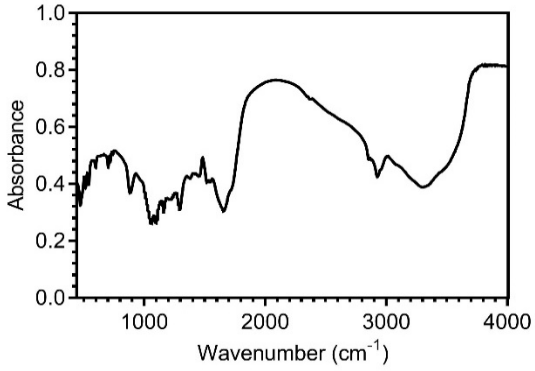

Supplementary Figure S2. Representative EPR spectra of HAp-7.

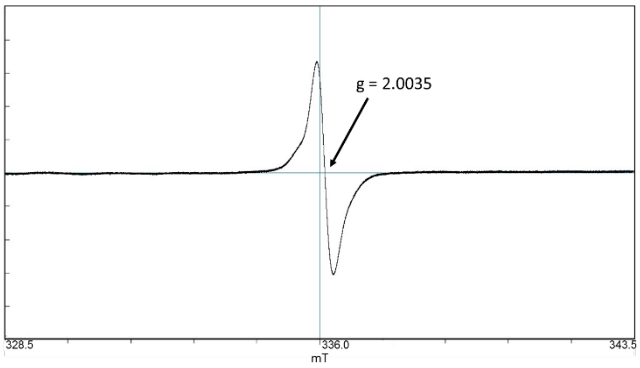

Supplement: Supplementary file 1 [file molecules-23-00753-s001.pdf]
